# Photoactivated chromophore-corneal cross-linking accelerates corneal healing in fungal keratitis: an updated meta-analysis

**DOI:** 10.1186/s13643-023-02380-5

**Published:** 2023-11-11

**Authors:** Shuyi Liu, Shifeng Fang, Lijun Zhang

**Affiliations:** 1 Graduate School, Dalian Medical University, Dalian, Liaoning Province, 116044 China; 2 Department of Ophthalmology, The Third People’s Hospital of Dalian, Non-Directly Affiliated Hospital of Dalian Medical University, Dalian, Liaoning Province, 116033 China

**Keywords:** PACK-CXL, Keratitis, Infection, Meta-analysis, Systematic review

## Abstract

**Aim:**

To determine the effectiveness and safety of photoactivated chromophore-corneal cross-linking (PACK-CXL) adjuvant in infectious keratitis by April 5, 2022.

**Methods:**

We searched randomized controlled trials (RCTs) comparing standard antibiotic treatment (SAT) plus PACK-CXL to SAT in infectious keratitis in Embase, MEDLINE with PubMed, Web of Science, and Cochrane Library. We independently screened and extracted data using predesigned tables. Cochrane’s risk-of-bias tool was utilized to examine the quality of RCTs. A random-effects model was employed to determine the overall effect size of the meta-analyses. Grading of Recommendations, and Assessment, Development and Evaluations (GRADE) was also performed to examine the quality of evidence.

**Results:**

Seven eligible RCTs with 283 patients were acquired. Adjuvant PACK-CXL reduced the time needed to perform corneal healing in fungal keratitis (− 1.33 months; 95% *CI*, − 1.83 to − 0.42, *I*^2^ = 0%, *P* < 0.05) as compared to SAT alone. The risks of adverse events were not significantly different both in fungal and bacterial keratitis. Due to the substantial heterogeneity among studies, such as population, the type and severity of infectious keratitis, drug regimens of SAT, PACK-CXL protocol, and the judgment of subjective outcomes, the evidence grade was low.

**Conclusion:**

Adjuvant PACK-CXL accelerates fungal keratitis healing as compared to SAT alone. But more rigorous RCTs are required to determine the clinical effectiveness and safety.

**Supplementary Information:**

The online version contains supplementary material available at 10.1186/s13643-023-02380-5.

## Introduction

Infectious keratitis is the fifth leading cause of blindness overall causing 3.5% (36 million) of all blind individuals up to 2015 [1]. The incidence of infectious keratitis differs worldwide [1]. It has been reported at 2.5 to 40.3 cases per 100,000 population/year in developed countries while as high as 113 to 799 cases per 100,000 population/year in developing countries [1–3]. Infectious keratitis can be caused by a wide variety of pathogens, including bacteria, fungi, viruses, and parasites such as acanthamoeba [2]. Because of the high morbidity and considerable societal burden, the prevention and treatment of infectious keratitis are crucial [3, 4]. Both antimicrobial treatment and surgical intervention, such as topical antimicrobial drug administration, amniotic membrane transplantation, and therapeutic penetrating keratoplasty, have been performed [5]. One novel intervention is the application of photoactivated chromophore for keratitis-corneal cross-linking (PACK-CXL) [6].

Ting et al. conducted a meta-analysis in 2019 that included forty-six studies (four RCTs) assessing PACK-CXL for infectious keratitis. When compared to SAT alone, PACK-CXL was characterized by a shortened mean duration for corneal healing and a quicker clearance of corneal infiltration [7]. Papaioannou et al. conducted a similar meta-analysis in 2016 which included twenty-five studies (including two RCTs). PACK-CXL seems promising in handling infectious keratitis excluding herpetic keratitis, with increased expectations for bacterial and acanthamoeba cases comparing with fungal keratitis [8]. Davis et al. conducted a meta-analysis in 2020 that included three trials (two RCTs and one quasi-RCT), which reached the opposite conclusion. It is very uncertain whether PACK-CXL with SAT is more effective than SAT alone for re-epithelialization and complete healing in bacterial keratitis [9]. Not only RCTs but also case reports, quasi-RCTs, and case series were eligible in these meta-analyses, so high heterogeneity across studies was inevitable. Thus, the application of PACK-CXL in infectious keratitis remains controversial [10]. Another four newly published RCTs, including 208 patients focused on the same topic, have been identified [11–14]. Therefore, a comprehensive review and meta-analysis including only RCTs were performed to determine the effectiveness and safety of an adjuvant PACK-CXL in infectious keratitis.

## Methods

### Protocol

This study was conducted in line with the Cochrane Handbook for Systematic Reviews of Interventions and the Preferred Reporting Items for Systematic Reviews and Meta-Analyses statement (PRISMA 2020; Supplementary materials) [15, 16].

### Literature search

Relevant articles, limited to human and RCTs in Embase, MEDLINE with PubMed, Web of Science, and Cochrane Library, were searched and published up to April 5, 2022, by two authors (S. Y. Liu and S. F. Fang). The search strategy contained three components: clinical condition (“keratitis,” “corneal ulcer”), intervention (“cross-linking reagents,” “riboflavin,” “anti-infective agents”, “ultraviolet therapy,” “photosensitizing agents,” “ultraviolet rays,” “collagen”), and study type (randomized clinical trial). Detailed search strategies are provided in the Supplementary materials. Further, the reference lists in the eligible RCTs were examined manually in case that there were other eligible studies. This procedure was repeated until no more studies were discovered.

#### Eligibility criteria

1. Participants: Patients suffering from infectious keratitis with confirmed diagnosis, encompassing bacterial and fungal cases
2. Intervention: Adjuvant PACK-CXL
3. Comparison: Standard antimicrobial treatment (SAT) alone
4. Outcomes
  Primary outcome: The duration of corneal healing performed, characterized as thorough corneal re-epithelialized and corneal infiltration and/or hypopyon eradication
  Secondary outcome: The size of corneal epithelial defect at 1 week, the size of corneal infiltrate at 1 week, the depth of corneal infiltrate at final follow-up, visual acuity (mean logarithm of the minimum angle of resolution) at final follow-up, and adverse events: worsening infectious keratitis and/or corneal melt requiring tectonic or therapeutic keratoplasty or evisceration at final follow-up (1 to 6 months).
5. Study type: Randomized controlled trials (RCTs)

#### Exclusion criteria

Individuals who received extra interventions except antimicrobial treatment which might accelerate corneal healing were excluded.

### Study selection

Based on predefined criteria, two authors (S. Y. Liu and S. F. Fang) independently selected the title and abstract of the 1256 studies identified by the search. Following that, we downloaded the full texts of these articles and conducted a review. Another author (L. J. Zhang) examined the data. Discussions were also performed if there was a divergence.

### Data extraction

Two authors (S. Y. Liu and S. F. Fang) independently extracted data in the included RCTs: first author, publication year, sample size, patient characteristics, the protocol of adjuvant PACK-CXL, drug regimen, and other outcomes data. Another author (L. J. Zhang) examined the data. Discussions were also performed if there was a divergence.

### Risk-of-bias assessment

Cochrane Collaboration’ s tool was utilized to identify the risk of bias [17]. Trials were scored as high, low, or unclear based on the following items: random sequence generation, allocation concealment, blinding of participants and personnel, blinding of outcome assessment, incomplete outcome data, selective reporting, and other bias. The study with high risk for ≧ 1 item was regarded as high risk, and the study with low risk for all items was regarded as low risk. Otherwise, the study was regarded as unclear risk [18].

### Data synthesis and analysis

Risk ratio (RR) with its 95% confidence interval (CI) for dichotomous outcome and mean difference (MD) with its 95% CI for continuous outcome were conducted. A random-effects model was utilized due to the clinical heterogeneity. Two-sided *P* < 0.05 was regarded as statistically significant. Statistical analyses were performed in RevMan 5.3 software.

### Certainty of evidence

Certainty of evidence was examined using Grading of Recommendations, and Assessment, Development and Evaluations (GRADE) [19, 20], and summary tables were obtained through GRADE profiler online (https://gradepro.org/).

## Results

### Trial selection

**Fig. 1:**
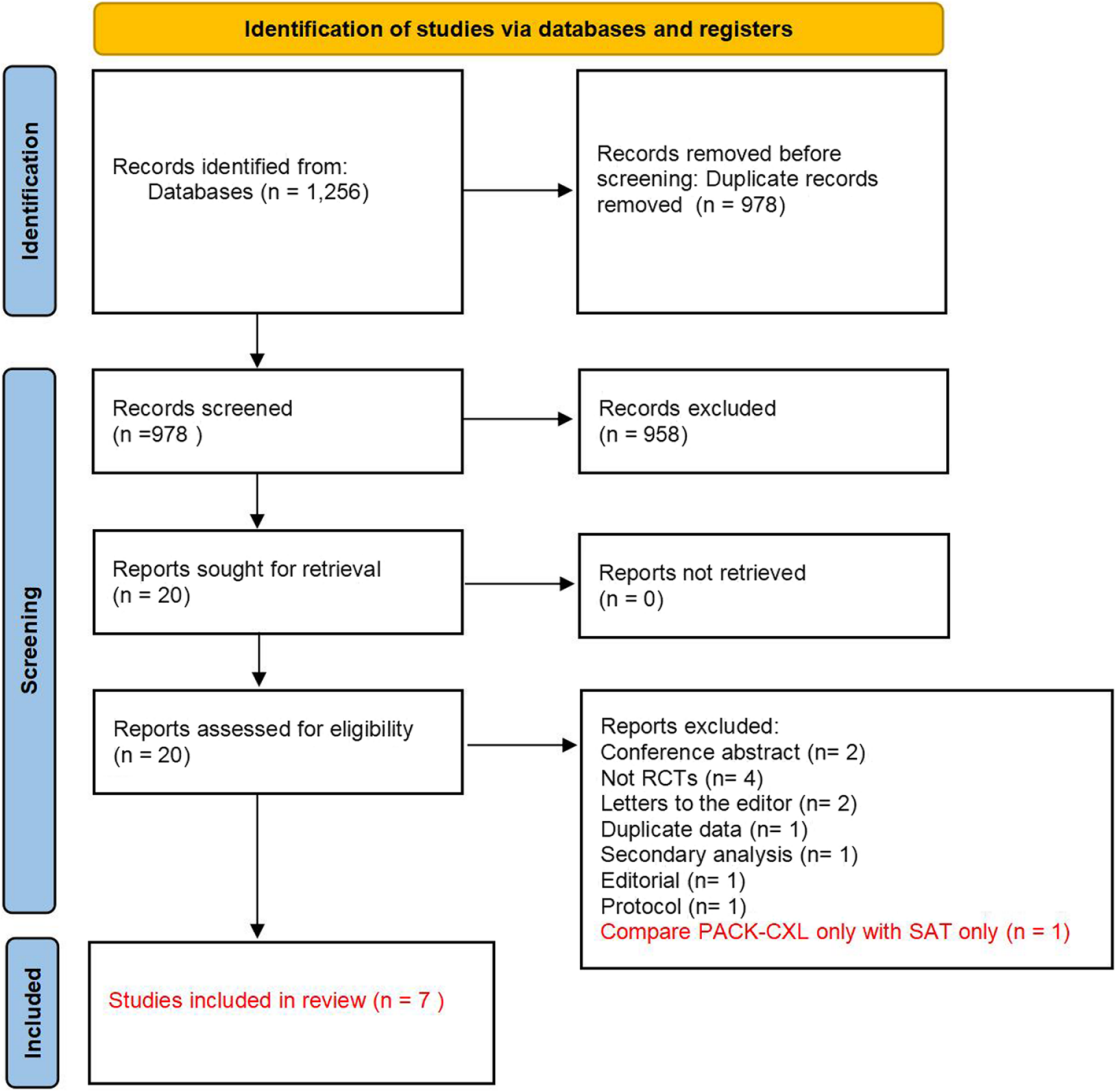
Literature screening flowchart

A total of 1256 studies were screened, in which 278 duplicate publications (22%) and 958 irrelevant studies (76%) were excluded after the initial screening. Full text of the twenty (2%) studies were acquired for further evaluation. Thirteen studies were excluded: two conference abstracts [21, 22], one editorial [23], one protocol [24], four studies without RCT design [25–28], two letters to the editor [29, 30], one study with duplicate data [31], one secondary analysis [32], one compares PACK-CXL only with SAT only [33]. Finally, seven RCTs were included [11–14, 34–36], and the procedure is shown in Fig. 1.

### Trial characteristics

The characteristics of these RCTs were shown in Table 1. The publication year ranged from 2015 to 2022, and the sample size ranged from 13 to 111 (together, 283). Eighty patients were diagnosised as bacterial keratitis, and 203 patients were diagnosised as fungal keratitis. One-hundred thirty-nine patients received adjuvant PACK-CXL and SAT, while 144 patients received SAT only. All the trials recorded adverse events [11–14, 34–36], three trials reported the duration of corneal healing performed [11, 13, 34], two trials reported the size of corneal epithelial defect at 1 week [34, 36], three trials reported the size of corneal infiltrate at 1 week [11, 34, 36], only one trial reported the depth of corneal infiltrate at final follow-up [11], and six trials reported visual acuity at final follow-up [11–14, 35, 36].

**Table 1:** Characteristics of studies included

Trial	Sample size (T/C)	Age, years (T/C)	Male gender (%)	Diagnosis	Follow-up
Bamdad et al. (2015) [34]	16/16	39.6 ± 16.8/40.3 ± 14.9	21 (66%)	BK	1 month
Uddaraju et al. (2015) [35]	6/7	39.5 (35–41)/56 (40–62)	8 (61%)	FK	6 weeks
Kasetsuwan et al. (2016) [36]	15/15	44.60 (17–73)/53.93 (15–84)	21 (70%)	BK/FK (12/18)	1 month
Wei et al. (2019) [11]	21/20	53.4 ± 2.65/54.75 ± 3.67	26 (63%)	FK	6 months
Prajna et al. (2020) [12]	55/56	55 (47, 65), 56 (45, 65)/50 (36, 55), 45 (40, 58)	63 (57%)	FK	3 months
Jeyalatha et al. (2021) [13]	9/11	49 ± 13.3/50 ± 9.1	16 (80%)	FK	1 month
Prajna et al. (2021) [14]	17/19	59 (48–66)/60 (54.5–65)	21 (58%)	BK	3 months
Trial	PACK-CXL treatment protocol		SAT regimen		
Bamdad et al. (2015) [34]	The same day before SAT	365 nm × 3 mW/cm^2^ × 30 min	Cefazolin, gentamicin		
Uddaraju et al. (2015) [35]	After SAT for 2 weeks	370 nm × 3 mW/cm^2^ × 30 min	Natamycin, voriconazole		
Kasetsuwan et al. (2016) [36]	The same day before SAT	365 nm × 3 mW/cm^2^ × 30 min	Cefazolin, amikacin; amphotericin B, natamycin		
Wei et al. (2019) [11]	The same day before SAT	365 nm × 3 mW/cm^2^ × 30 min	Natamycin, voriconazole		
Prajna et al. (2020) [12]	Within 24 h of enrollment	365 nm × 3 mW/cm^2^ × 30 min	Natamycin, voriconazole		
Jeyalatha et al. (2021) [13]	Not reported	370 nm × 3 mW/cm^2^ × 30 min, 5.4 J/cm^2^	Natamycin, voriconazole/amphotericin B		
Prajna et al. (2021) [14]	Within 24 h of enrollment	365 nm × 3 mW/cm^2^ × 30 min	Moxifloxacin		

Bacterial keratitis, *BK*; fungal keratitis, *FK*; *T*, PACK-CXL plus SAT; *C*, SAT

### Risk of bias

**Fig. 2:**
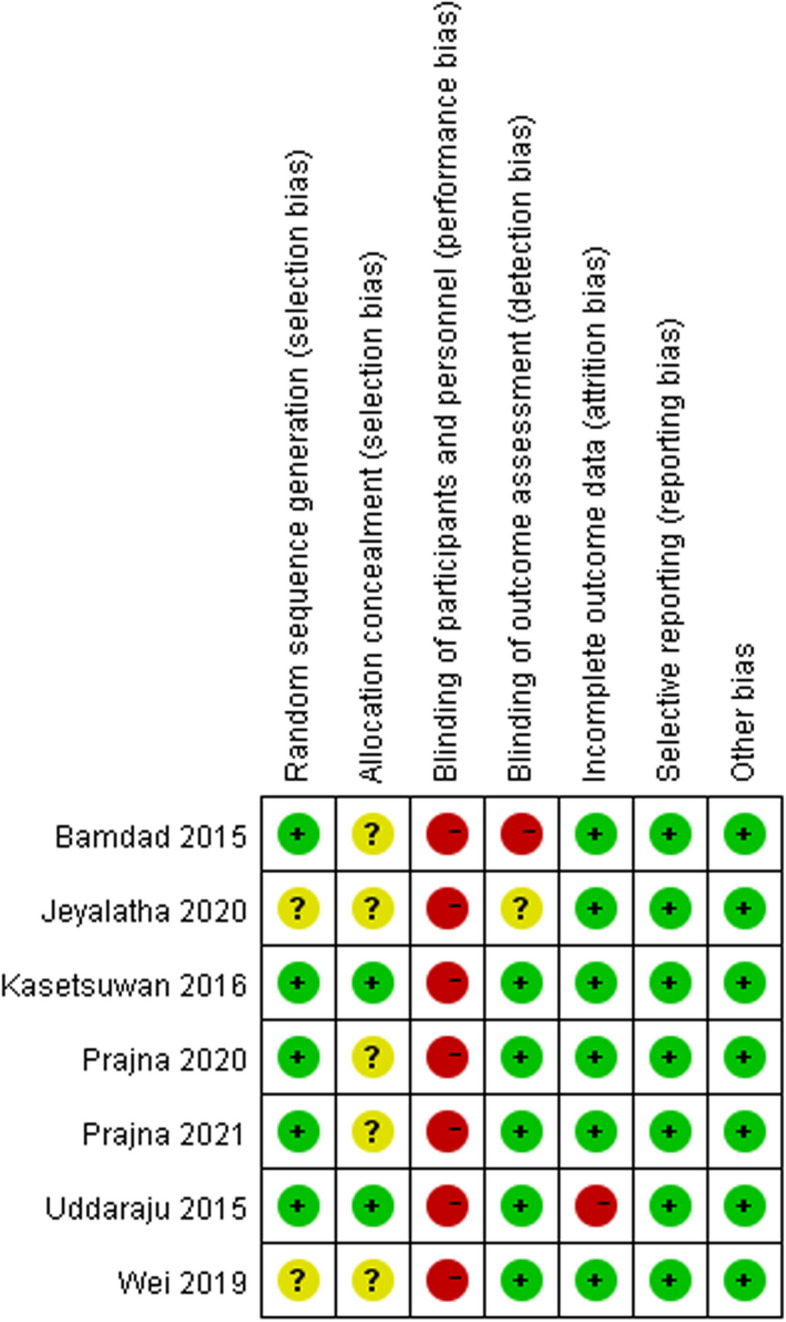
Risk of bias. + , low; ?, uncertain; -, high

Risk-of-bias assessment for the seven trials was presented in Fig. 2. All the trials were classified as with a high risk of bias.

### The duration of corneal healing performed

**Fig. 3:**
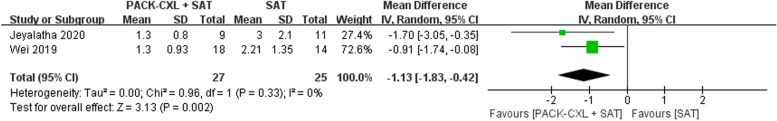
Forest plot for the duration of corneal healing performed

Fifty-two patients with fungal keratitis in two trials were included (27 patients with adjuvant PACK-CXL) [11, 13]. Thirty-two patients with bacterial keratitis in one trial were included (16 patients with adjuvant PACK-CXL) [34]. Adjuvant PACK-CXL significantly reduced the time needed to perform corneal healing in fungal keratitis (*MD* =  − 1.13, 95% *CI*, − 1.83 to − 0.42, *P* < 0.05), with low heterogeneity among the two trials (*I*^2^ = 0%, *P* = 0.33) (Fig. 3).

### The size of corneal epithelial defect (mm^2^) at 1 week

Eighteen patients with fungal keratitis in one trial were included (eight patients with adjuvant PACK-CXL) [36]. Forty-four patients with bacterial keratitis in two trials were included (23 patients with adjuvant PACK-CXL) [34, 36]. All these trials found that adjuvant PACK-CXL could not reduced the size of corneal epithelial defect at 1 week in fungal and bacterial keratitis. However, Kasetsuwan et al. presented this outcome with “median, quartile,” and we failed to perform a meta-analysis.

### The size of corneal infiltrate (mm^2^) at 1 week

Fifty-one patients with fungal keratitis in two trials were included (29 patients with adjuvant PACK-CXL) [11, 36]. Fifty-four patients with bacterial keratitis in two trials were included (23 patients with adjuvant PACK-CXL) [34, 36]. For fungal keratitis, all these trials found that adjuvant PACK-CXL could not reduced the size of corneal infiltrate at 1 week. For bacterial keratitis, it was controversial. Bamdad et al. found that adjuvant PACK-CXL could reduced the size of corneal infiltrate at 1 week, while Kasetsuwan et al. found that it could not. However, Kasetsuwan et al. presented this outcome with “median, quartile,” and we failed to perform a meta-analysis.

### The depth of corneal infiltrate (μm) at final follow-up

Only one trial was included; the ulcer depth did not reduce with the administration of PACK-CXL (*P* > 0.05) [11]. Therefore, analysis was not carried out.

### Visual acuity (mean logarithm of the minimum angle of resolution) at final follow-up

Two-hundred three patients with fungal keratitis in five trials were included (99 patients with adjuvant PACK-CXL) [11–13, 35, 36]. Forty-eight patients with bacterial keratitis in two trials were included (24 patients with adjuvant PACK-CXL) [14, 36]. All these trials found that adjuvant PACK-CXL could not improve visual acuity, even might result in decreased visual acuity. However, some RCTs presented this outcome with “median, quartile,” and we failed to perform a meta-analysis.

### Adverse events

**Fig. 4:**
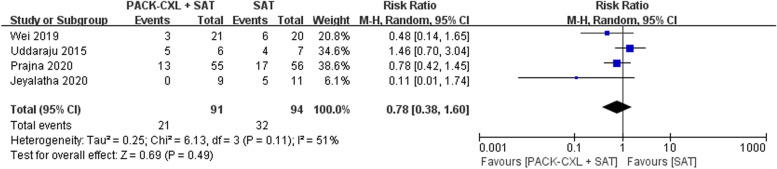
Forest plot for adverse events in fungal keratitis at the last follow-up

**Fig. 5:**
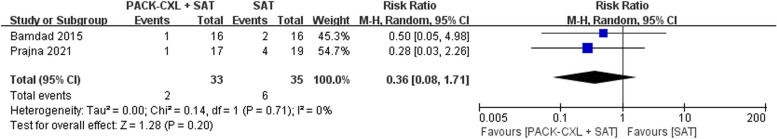
Forest plot for adverse events in bacterial keratitis at the last follow-up

One-hundred eighty-five patients with fungal keratitis in four trials were included (91 patients with adjuvant PACK-CXL) [11–13, 35]. Sixty-eight patients with bacterial keratitis in one trial were included (33 patients with adjuvant PACK-CXL) [14, 34]. Adjuvant PACK-CXL could not reduce adverse events in both fungal and bacterial keratitis (*RR* = 0.78, 95% *CI*, 0.38 to 1.60, *P* = 0.49; *RR* = 0.36, 95% *CI*, 0.08 to 1.71, *P* = 0.20, respectively) (Figs. 4 and 5).

### Quality of evidence

GRADE evidence is presented in Table 2. In terms of the duration of corneal healing performed, and adverse events risk, the certainty of evidence was low.

**Table 2:** Quality of evidence

Certainty assessment							No. of patients		Effect		Certainty	Importance
No. of studies	Study design	Risk of bias	Inconsistency	Indirectness	Imprecision	Other considerations	PACK-CXL + SAT	SAT	Relative (95% *CI*)	Absolute (95% *CI*)		
**The duration of corneal healing performed**												
2	Randomized trials	Serious^a^	Not serious	Not serious	Serious^b^	None	27	25	-	MD **1.13 lower** (0.183 lower to 0.42 lower)	⨁⨁◯◯ Low	Critical
**Adverse events in fungal keratitis**												
4	Randomized trials	Serious^a^	Not serious	Not serious	Serious^b^	None	21/91 (23.1%)	32/94 (34.0%)	***RR* 0.78** (0.38 to 1.60)	**75 fewer per 1000** (from 211 fewer to 204 more)	⨁⨁◯◯ Low	Critical
**Adverse events in bacterial keratitis**												
2	Randomized trials	Serious^a^	Not serious	Not serious	Serious^b^	None	2/33 (6.1%)	6/35 (17.1%)	***RR* 0.36** (0.08 to 1.71)	**110 fewer per 1000** (from 158 fewer to 122 more)	⨁⨁◯◯ Low	Critical

*CI* confidence interval, *MD* mean difference, *RR* risk ratio

Explanations: ^a^Risk of bias, ^b^Small sample

## Discussion

### Main findings

We reviewed the relevant studies that compared the effectiveness and safety of adjuvant PACK-CXL versus SAT alone in infectious keratitis comprehensively and systematically. Adjuvant PACK-CXL reduced the time needed to perform corneal healing as compared to SAT alone in fungal keratitis. The risks of adverse events were not significantly different in both fungal and bacterial keratitis.

### Compared with published literature

For the primary outcome, the duration of corneal healing performed, two studies on this subject have been published but reached the opposite conclusion. Davis et al. revealed that it was very uncertain whether adjuvant PACK-CXL was more effective than SAT alone for reepithelialization and complete healing [9]. While Ting et al. found that when compared to SAT alone, adjuvant PACK-CXL resulted in shorter mean time to complete corneal healing [7]. Different types of infectious keratitis may be the underlying reason for this divergence. Therefore, we conducted a subgroup analysis based on infection type and revealed that adjuvant PACK-CXL could reduce the time needed to perform corneal healing as compared to SAT alone in fungal keratitis. For the risks of adverse effects, we also conducted subgroup analyses based on infection type. In accordance with the previous study, adjuvant PACK-CXL could not reduce the risks of adverse effects as compared to SAT alone in both bacterial and fungal keratitis [7].

Certain differences should be highlighted. Firstly, previous studies included case reports, quasi-RCTs, and case series. To achieve reliable estimates, we set strict inclusion criteria. Only the RCTs clarified clearly the enrollment of patients with adjuvant PACK-CXL were included. We further included another four newly published RCTs with 208 patients published in 2019–2021 in this meta-analysis, which promoted statistical power [11–14]. Secondly, we conducted subgroup analyses based on infection type. Thirdly, due to clinical heterogeneity, a random-effects model was chosen to guarantee a more conservative estimation. Lastly, the GRADE method was employed to evaluate the certainty of evidence in order to assist clinical practice. Therefore, the present study was the most updated and thorough, reinforcing prior results.

### Clinical practice implication

In recent years, the antimicrobial effect of PACK-CXL has been investigated in infectious keratitis [37, 38]. However, the administration of PACK-CXL in infectious keratitis was still controversial [39, 40].

We analyzed the suspected reasons, and possible explanations are as follows. Firstly, the clinical outcomes and the risk of adverse events may be related to the severity of infectious keratitis. Uddaraju et al. enrolled patients with culture-positive deep stromal fungal keratitis who had not responded to appropriate treatment for 2 weeks [35]. The poor response could be explained that fungal infections penetrated deeper, and the intensity of UV-A light would not be sufficient to treat. Early and superficial fungal keratitis responded well to PACK-CXL [27, 41]. While in advanced and deep stromal fungal keratitis, it was hard to determine whether the infection reacted to PACK-CXL alone [42]. Secondly, drug regimens of SAT were not uniform in the seven eligible trials. Thirdly, although most of the included studies used the standard Dresden protocol that UV-A radiation exposure of 3 mW/cm^2^ for 30 min, the lengths of wave were different, 365 nm or 370 nm. The efficacy of PACK-CXL followed the Bunsen-Roscoe law of reciprocity, and higher fluence or irradiance substantially increased the killing rates [33, 43], while some study suggested that accelerated PACK-CXL provided an antimicrobial effect similar to the low-intensity, slow setting [44]. Therefore, safety limits for clinical application are required in further studies. Lastly, different causative microorganisms were included in this study. Alio et al. revealed that PACK-CXL decreased corneal melting with the following order from most to least: gram-negative bacteria, gram-positive bacteria, acanthamoeba, and fungus [45]. It was worth noting that PACK-CXL presented a weaker killing effect in acanthamoeba, and even be a risk of activating the latent virus, so PACK-CXL should be applied carefully, in patients with acanthamoeba or viral keratitis [46, 47]. In short, ophthalmologists should pay more attention to the type and severity of infectious keratitis, drug regimens of SAT, and PACK-CXL protocol in clinical practice.

### Strengths and limitations

The strength of the present study was that only RCTs were included to reduce heterogeneity. And GRADE method was applied to examine the certainty of evidence. There were some limitations in the present study which may affect the interpretation. Firstly, all the included trials presented detailed data; however, different trials focused on different outcomes or documented the same outcome at different follow-up time. So, it was hard to synthesize the data and perform meta-analysis. Secondly, since only seven trials were included, it was difficult to deny the heterogeneity between the included studies. The heterogeneity may come from the following: population, the type and severity of infectious keratitis, drug regimens of SAT, PACK-CXL protocol, and the judgment of subjective outcomes. All these factors may lead to the lack of precise proof to recommend PACK-CXL as a potential approach in infectious keratitis. For further RCTs trials, we suggested that the type and severity of infectious keratitis, drug regimens of SAT, and PACK-CXL protocol should be in the consistency, making the outcomes be more comparable. Meanwhile, the judgment of subjective outcomes should be performed by at least two ophthalmologists, making the data more precise. Therefore, more RCTs comparing cases with similar baseline are needed.

## Conclusions

The present study suggests that adjuvant PACK-CXL accelerates corneal healing in fungal keratitis compared with SAT alone. Ophthalmologists should pay more attention to the type and severity of infectious keratitis, drug regimens of SAT, and PACK-CXL protocol in clinical practice. And more rigorous trials are needed in the future.

### Supplementary Information

**Additional file 1: Supplementary material 1: Appendix 1.** Details of the Literature Search Strategy.

**Additional file 2: Supplementary material 2. **PRISM 2020 Checklist.

**Additional file 3: Supplementary material 3.** Data extraction.

**Additional file 4: Supplementary material 4.** Details of adverse events.

**Additional file 5: Supplementary material 5.** PICO.

**Additional file 6: Supplementary material 6.** PACK-CXL for infectious keratitis.

## Data Availability

The data sets in the study are presented in the article or supplementary material, and further information can be directed to the authors.
