# Supplementary material for: Photoactivated chromophore-corneal cross-linking accelerates corneal healing in fungal keratitis: an updated meta-analysis
Source: Syst Rev. 2023 Nov 11;12:208. doi: 10.1186/s13643-023-02380-5 (PMC10638714; doi:10.1186/s13643-023-02380-5)
Supplement: Supplementary file 1 — Additional file 1: Supplementary material 1: Appendix 1. Details of the Literature Search Strategy. [file 13643_2023_2380_MOESM1_ESM.docx]

**Appendix Ⅰ**

**Details of the Literature Search Strategy**

(1) PubMed （1977 to April 5, 2022）

| **Search** | **Query** | **Items found** |
| --- | --- | --- |
| #1 | "Corneal Ulcer"[Mesh] | 5,421 |
| #2 | "Keratitis"[MeSH Terms] | 21,864 |
| #3 | "keratitis*"[Title/Abstract] OR "keratoconjunctivitis*"[Title/Abstract] OR "corneal ulcer*"[Title/Abstract] OR "corneal infect*"[Title/Abstract] | 23,859 |
| #4 | #1 OR #2 OR #3 | 30,718 |
| #5 | "Cross-Linking Reagents"[MeSH Terms] | 26,519 |
| #6 | "cross link*"[Title/Abstract] OR "crosslink*"[Title/Abstract] OR "cross link*"[Title/Abstract] OR "cxl"[Title/Abstract] | 116,540 |
| #7 | "Collagen"[MeSH Terms] | 128,824 |
| #8 | "collagen*"[Title/Abstract] | 232,933 |
| #9 | #5 OR #6 OR #7 OR #8 | 370,845 |
| #10 | "Anti-Infective Agents"[MeSH Terms] | 784,601 |
| #11 | "anti infective agents"[Title/Abstract] OR "anti infective agent*"[Title/Abstract] OR "microbicide*"[Title/Abstract] | 4,696 |
| #12 | #10 OR #11 | 785,993 |
| #13 | "Riboflavin"[MeSH Terms] | 15,085 |
| #14 | "riboflavin*"[Title/Abstract] OR "vitamin b*"[Title/Abstract] | 49,300 |
| #15 | "Photosensitizing Agents"[MeSH Terms] | 19,692 |
| #16 | "photosensiti*"[Title/Abstract] | 33,254 |
| #17 | "Ultraviolet Therapy"[MeSH Terms] | 8,961 |
| #18 | ("ultraviolet"[All Fields] OR "ultraviolets"[All Fields]) AND "therapy*"[All Fields]  Translations  Ultraviolet: "ultraviolet"[All Fields] OR "ultraviolets"[All Fields] | 16,066 |
| #19 | "Ultraviolet Rays"[MeSH Terms] | 81,336 |
| #20 | "ultraviolet*"[Title/Abstract] OR "ultra violet*"[Title/Abstract] OR "uv"[Title/Abstract] OR "actinic ray*"[Title/Abstract] | 232,550 |
| #21 | #13 OR #14 OR #15 OR #16 OR #17 OR #18 OR #19 OR #20 | 353,133 |
| #22 | #9 OR #12 OR #21 | 1,478,779 |
| #23 | #22 AND #4 | 6,177 |
| #24 | "randomized controlled trial"[Title/Abstract] OR "randomized"[Title/Abstract] | 605,761 |
| #25 | #24 AND #23 | 187 |

(2) Embase (1974 to April 6, 2022)

| **Search** | **Query** | **Items found** |
| --- | --- | --- |
| #1 | 'keratitis'/exp | 38,779 |
| #2 | 'cornea ulcer'/exp | 9,210 |
| #3 | keratitis* OR keratoconjunctivitis*:ab,ti OR 'corneal ulcer*':ab,ti OR 'corneal infect*':ab,ti | 36,718 |
| #4 | #1 OR #2 OR #3 | 43,562 |
| #5 | 'cross linking reagent'/exp | 6,580 |
| #6 | 'cross link*' OR crosslink*:ab,ti OR 'cross link*':ab,ti OR cxl*:ab,ti | 151,438 |
| #7 | 'collagen'/exp | 244,945 |
| #8 | collagen* | 384,076 |
| #9 | 'antiinfective agent'/exp | 4,376,998 |
| #10 | 'anti-infective agent*' OR 'anti infective agent*':ab,ti OR microbicide*:ab,ti | 5,898 |
| #11 | 'riboflavin'/exp | 21,350 |
| #12 | riboflavin* OR 'vitamin b*':ab,ti | 66,339 |
| #13 | 'photosensitizing agent'/exp | 56,525 |
| #14 | photosensiti* OR 'photosensitizing agent*':ab,ti | 56,263 |
| #15 | 'ultraviolet phototherapy'/exp | 11,285 |
| #16 | 'ultraviolet therapy*' | 185 |
| #17 | 'ultraviolet radiation'/exp | 135,935 |
| #18 | ultraviolet* OR 'ultra violet*':ab,ti OR uv*:ab,ti OR 'actinic ray*':ab,ti | 417,660 |
| #19 | #5 OR #6 OR #7 OR #8 OR #9 OR #10 OR #11 OR #12 OR #13 OR #14 OR #15 OR #16 OR #17 OR #18 | 5,256,985 |
| #20 | 'randomized controlled trial'/exp | 707,647 |
| #21 | 'randomized*' | 1,281,902 |
| #22 | #20 OR #21 | 1,282,758 |
| #23 | #4 AND #19 AND #22 | 789 |

(3) Cochrane Library (April 6, 2022)

| **Search** | **Query** | **Items found** |
| --- | --- | --- |
| #1 | MeSH descriptor: [Keratitis] explode all trees | 1058 |
| #2 | MeSH descriptor: [Corneal Ulcer] explode all trees | 167 |
| #3 | (keratitis*):ti,ab,kw OR (Keratoconjunctivitis*):ti,ab,kw OR ("Corneal ulcer*"):ti,ab,kw OR ("Corneal infect*"):ti,ab,kw | 2226 |
| #4 | #1 OR #2 OR #3 | 2226 |
| #5 | MeSH descriptor: [Cross-Linking Reagents] explode all trees | 181 |
| #6 | (Cross-Link*):ti,ab,kw OR (CrossLink*):ti,ab,kw OR ("Cross Link*"):ti,ab,kw OR (CXL*):ti,ab,kw | 2237 |
| #7 | MeSH descriptor: [Collagen] explode all trees | 2589 |
| #8 | (Collagen*):ti,ab,kw | 9318 |
| #9 | ("Anti-Infective Agents"):ti,ab,kw OR ("Anti Infective Agent*"):ti,ab,kw OR (Microbicide*):ti,ab,kw | 5450 |
| #10 | MeSH descriptor: [Anti-Infective Agents] explode all trees | 31146 |
| #11 | (Riboflavin*):ti,ab,kw OR ("Vitamin B*"):ti,ab,kw | 3371 |
| #12 | MeSH descriptor: [Riboflavin] explode all trees | 439 |
| #13 | (Photosensiti*):ti,ab,kw | 1854 |
| #14 | MeSH descriptor: [Photosensitizing Agents] explode all trees | 809 |
| #15 | MeSH descriptor: [Ultraviolet Therapy] explode all trees | 634 |
| #16 | (Ultraviolet Therapy*):ti,ab,kw | 1681 |
| #17 | MeSH descriptor: [Ultraviolet Rays] explode all trees | 704 |
| #18 | (Ultraviolet*):ti,ab,kw OR (Ultra-violet*):ti,ab,kw OR (UV*):ti,ab,kw OR ("Actinic Ray*"):ti,ab,kw | 8098 |
| #19 | #5 OR #6 OR #7 OR #8 OR #9 OR #10 OR #11 OR #12 OR #13 OR #14 OR #15 OR #16 OR #17 OR #18 | 53794 |
| #20 | ("randomized-controlled trial"):ti,ab,kw OR (randomized):ti,ab,kw | 987858 |
| #21 | #4 AND #19 AND #20 | 219 |

(4) [Web of Science](https://apps.webofknowledge.com/home.do?SID=6BQQjiiMCVa9MgFvRpC) core collection (1986 to April 6, 2022)

| **Search** | **Query** | **Items found** |
| --- | --- | --- |
| #1 | Keratoconjunctivitis* (topic) or keratitis* (topic) or "Corneal ulcer* " (topic) or "Corneal infect*" (topic) | 25,479 |
| #2 | Cross-Link* (topic) or CrossLink* (topic) or "Cross Link* " (topic) or CXL* (topic) | 254,125 |
| #3 | Collagen* (topic) | 285,061 |
| #4 | "Anti-Infective Agent*" (topic) or "Anti Infective Agent* " (topic) or Microbicide* (topic) | 5,345 |
| #5 | Riboflavin* (topic) or "Vitamin B*" (topic) | 55,644 |
| #6 | Photosensiti* (topic) | 67,151 |
| #7 | Ultraviolet Therapy* (topic) | 5,446 |
| #8 | randomized* (topic) or "randomized controlled trial*" (topic) | 852,085 |
| #9 | Ultraviolet Ray (topic) or Ultra-violet (topic) or UV (topic) or "Actinic Ray*" (topic) | 556,343 |
| #10 | #2 OR #3 OR #4 OR #5 OR #6 OR #7 OR #9 | 1,175,506 |
| #11 | #10 AND #8 AND #1 | 61 |
