# Supplementary material for: Photoactivated chromophore-corneal cross-linking accelerates corneal healing in fungal keratitis: an updated meta-analysis
Source: Syst Rev. 2023 Nov 11;12:208. doi: 10.1186/s13643-023-02380-5 (PMC10638714; doi:10.1186/s13643-023-02380-5)
Supplement: Supplementary file 4 — Additional file 4: Supplementary material 4. Details of adverse events. [file 13643_2023_2380_MOESM4_ESM.docx]

Adverse events [1-2]: worsening infectious keratitis and/or corneal melt requiring tectonic or therapeutic keratoplasty or evisceration at final follow-up (one to six months).

| Trial | Details of adverse events |
| --- | --- |
| Bamdad et al., 2015 | T: 1 patient required AMT  C: 1 patient required AMT and 1 patient required a conjunctival flap |
| Uddaraju et al., 2015 | T: 4 patients presented corneal perforation and 1 patient presented an increased infiltrate size  C: 4 patients presented an increased infiltrate size |
| Kasetsuwan et al., 2016 | T: 2 patients presented corneal perforation and 1 patient presented evisceration  C: 3 patients presented corneal perforation and 1 patient presented evisceration |
| Wei et al., 2019 | T: 3 patients presented corneal perforation and 9 patients required surgery (therapeutic keratoplasty or median tarsorrhaphy)  C: 6 patients presented corneal perforation and 11 patients required surgery (therapeutic keratoplasty or median tarsorrhaphy) |
| Prajna et al., 2020 | T: 4 patients presented corneal perforation  C: 6 patients presented corneal perforation |
| Jeyalatha et al., 2020 | T: none  C: 5 patients presented worsened infectious keratitis |
| Prajna et al., 2021 | T: 1 patient required therapeutic keratoplasty  C: 3 patients required therapeutic keratoplasty and 1 patient presented corneal perforation |

Note: T: PACK-CXL plus SAT; C: SAT; AMT: amniotic membrane transplantation;

Reference:

1. Ting Darren Shu Jeng,Henein Christin,Said Dalia G et al. Effectiveness of adjuvant photoactivated chromophore corneal collagen cross-linking versus standard antimicrobial treatment for infectious keratitis: a systematic review protocol.[J] .JBI Evid Synth, 2020, 18: 194-199.
2. Ting Darren Shu Jeng,Henein Christin,Said Dalia G et al. Photoactivated chromophore for infectious keratitis - Corneal cross-linking (PACK-CXL): A systematic review and meta-analysis.[J] .Ocul Surf, 2019, 17: 624-634.
