# Supplementary material for: Photoactivated chromophore-corneal cross-linking accelerates corneal healing in fungal keratitis: an updated meta-analysis
Source: Syst Rev. 2023 Nov 11;12:208. doi: 10.1186/s13643-023-02380-5 (PMC10638714; doi:10.1186/s13643-023-02380-5)
Supplement: Supplementary file 5 — Additional file 5: Supplementary material 5. PICO. [file 13643_2023_2380_MOESM5_ESM.docx]

| Participants | Patients suffering from infectious keratitis with confirmed diagnosis, encompassing bacterial and fungal cases. |
| --- | --- |
| Intervention | Adjuvant PACK-CXL. There is no restriction on the treatment protocol, including the regime of riboflavin administration, and the duration and intensity of the UV irradiation. |
| Comparison | Standard antimicrobial treatment (SAT) alone. There is no restriction on the treatment regime of standard topical antimicrobial treatment as it will vary significantly across different studies. For studies with no comparator, the effect of intervention will be presented in a narrative manner and will not be included in the meta-analysis. |
| Outcomes | Primary outcome: the duration of corneal healing performed, characterized as thorough corneal re-epithelialized and corneal infiltration and/ or hypopyon eradication.  Secondary outcome:   1. the size of corneal epithelial defect at one-week; 2. the size of corneal infiltrate at one-week; 3. the depth of corneal infiltrate at final follow-up; 4. visual acuity (mean Logarithm of the Minimum Angle of Resolution) at final follow-up; 5. adverse events: worsening infectious keratitis and/or corneal melt requiring tectonic or therapeutic keratoplasty or evisceration at final follow-up (one to six months). |
| Study type | Randomized controlled trials (RCTs). Only RCTs will be included in meta-analysis. |
